# The Impact of Robotic Rehabilitation on the Motor System in Neurological Diseases. A Multimodal Neurophysiological Approach

**DOI:** 10.3390/ijerph17186557

**Published:** 2020-09-09

**Authors:** Zoltán Zsigmond Major, Calin Vaida, Kinga Andrea Major, Paul Tucan, Gábor Simori, Alexandru Banica, Emanuela Brusturean, Alin Burz, Raul Craciunas, Ionut Ulinici, Giuseppe Carbone, Bogdan Gherman, Iosif Birlescu, Doina Pisla

**Affiliations:** 1 Research Center for Advanced Medicine “MedFuture”, University of Medicine and Pharmacy “Iuliu Hatieganu” Cluj-Napoca, 400000 Cluj-Napoca, Romania; zoltan.major@eeg-emg.ro; 2 Neurology Department, Municipal Clinical Hospital Cluj-Napoca, 400139 Cluj-Napoca, Romania; simorigabor@gmail.com (G.S.); emabrusturean@yahoo.com (E.B.); raul_rullz@yahoo.com (R.C.); 3 Research Center for Industrial Robots Simulation and Testing, Technical University of Cluj-Napoca, 400114 Cluj-Napoca, Romania; calin.vaida@mep.utcluj.ro (C.V.); paul.tucan@mep.utcluj.ro (P.T.); alexandru.banica@omt.utcluj.ro (A.B.); alin.burz@mep.utcluj.ro (A.B.); ionut.ulinici@omt.utcluj.ro (I.U.); giuseppe.carbone@unical.it (G.C.); bogdan.gherman@mep.utcluj.ro (B.G.); iosif.birlescu@mep.utcluj.ro (I.B.); 4 Second ICU, Neurosurgery Department, Cluj County Emergency Clinical Hospital, Strada Clinicilor 3–5, 400000 Cluj-Napoca, Romania; 5 DIMEG, University of Calabria, Via Pietro Bucci, 87036 Arcavacata, Italy

**Keywords:** robotic rehabilitation, physical therapy, stroke, Parkinson’s, ALS, qEEG, motor conduction time, turn-amplitude analysis

## Abstract

Motor disability is a key feature of many neurological diseases, influencing the social roles of affected patients and their ability to perform daily life activities. Current rehabilitation capacities are overwhelmed by the age-related increase of motor dysfunctions seen, for example, in stroke, extrapyramidal or neuromuscular diseases. As the patient to rehabilitation personnel ration increases, robotic solutions might establish the possibility to rapidly satisfy the increasing demand for rehabilitation. This paper presents an inaugural exploratory study which investigates the interchangeability of a novel experimental robotic rehabilitation device system with classical physical therapy, using a multimodal neurophysiological assessment of the motor system—quantitative electroencephalogram (EEG), motor conduction times and turn/amplitude analysis. Preliminary results show no significant difference between the two methods; however, a significant effect of the therapy was found on different pathologies (beneficial for vascular and extrapyramidal, or limited, and only on preventing reduction of joint movements in neuromuscular).

## 1. Introduction

The majority of neurological diseases also affect the motor system, causing disabilities incompatible with the person’s normal social role. Some of these have a known, clear etiology and therapy; for others, we are only at the beginning of the road, considering here both pathogenesis and treatment. When the motor system is involved, and equally so if the pyramidal or other parts of its architecture are involved, a key target is to rapidly overcome the deficit. Standardized medical protocols help to a point, but physical therapy has also a crucial role, regardless of the topography of the lesion. For example, stroke causes central motor neuron loss; motor neuron diseases affect both central and peripheral segments of the motor pathways; movement disorders, in particular Parkinson’s disease, influence modulation of the motor activity [1].

Rehabilitation programs are designed to stimulate plasticity of the involved pathways, supporting the recovery of motor capacities [2]. Whether areas of the motor cortex are disturbed by a vascular lesion-stroke [3], or by neurodegeneration, like in motor neuron diseases [4], physical rehabilitation influences the motor abilities of the patient. For stroke, a clear, quantifiable effect is measured and the approach became a standard for rehabilitation [5], both with classical physical therapy [6] and robotic assisted rehabilitation [7]. Motor neuron disease progresses regardless the therapeutic approach. Physical therapy, mainly assistive methods, in addition to robotic measures [8], seem to be beneficial in reducing the velocity of degradation [9]. Parkinson’s disease patients benefit from multidisciplinary rehabilitation [10], physical therapy being only one modality [11]. Gait, balance and standing are favorably enhanced by physical therapy [12], with robotic approaches being also widespread [13,14] for the same purposes. Rehabilitation protocols are available [15,16] and might be implemented both by human therapists or robotic devices [17].

An effective physical therapy program requires exercises several times a day, a demanding task for the healthcare system. The demographic evolution of the population imposes a change in the rehabilitation protocol paradigm; in the future, the patient/therapist ratio will become even more destabilized, with the ageing of the population. It is particularly important to provide solutions for the physical therapist to be able to work with multiple patients in the same time. Since the physical therapy often uses stereotyped, repetitive movements, the therapy may also be executed with the help of robotic devices. A physical therapist may only initiate, or program, and supervise such devices. One therapist is able to carry out several programs, concomitantly easing the increasing lack of healthcare personnel. The need for such devices was formulated in neurology wards also in our region, and, as an answer, ASPIRE [18,19] and ParReEx [20,21], both using a common, central controlling interface, were developed by the Technical University of Cluj-Napoca, designed for the upper limb rehabilitation.

Each robotic module can be operated separately with a distinct functionality, but also can work with three different patients simultaneously. Being a modular architecture, even if one module is not working, or a patient does not fit a module (for example a patient with arthrodesis of the elbow), the other modules still work and remain useful despite the technical or patient-dependent failure. The device was developed in close collaboration with the Neurology Department of the Municipal Clinical Hospital Cluj-Napoca. The collaboration was not limited to the clinical testing of the devices, but started with previous clinical activities which led the group to the development of a structure which is acceptable in clinical settings; in addition, the team of neurologists and physical therapists developed a database, which offers a safety interval in terms of forces and ranges of motion for the robotic devices. A functional prototype was developed, and a first exploratory clinical evaluation was started at the above-mentioned hospital, by a multidisciplinary team of neurologists, physical therapists, robotic engineers and nurses. The scope of this evaluation was on one hand to assess the robot’s performance and suitability in different clinical settings, and also to compare the effects of physical therapy carried out by the device with the exercises performed with the help of therapists. An exploratory, multimodal evaluation was conducted on three groups of patients, covering stroke, Parkinson’s disease and motor neuron disease. In the literature, different neurophysiological methods were used and proposed as quantitative measures, to evaluate the integrity of the whole architecture of the motor system. For stroke, there are studies even demonstrating the higher efficiency of robotic approach using neurophysiological methods by helping in the process of “rewiring” interhemisferic connectivity [22]. All these pathologies are presented below from the point of view of neurophysiological characterization.

Stroke. In case of stroke patients, central lesions are satisfactorily described by spectral expression of EEG slowing [23,24] and influences on motor evoked potentials [25]. The latter is even proposed as a predictive measure for recovery [26,27]. If the corticospinal pathway retains some of its integrity, recovery is sensibly characterized by measuring conductibility [28]; if this is enhanced by the rehabilitation method, it even might constitute an objective measure for efficiency [29]. Connectivity measures might also be considered as a predictor of recovery [30]. For the impact of upper motor neuron lesions on the activity of the lower motor neuron, there are only sporadic electromyogram (EMG) data in the literature [31]; still, turn-amplitude analysis shows influences, with changes over the rehabilitation process [32,33,34].

Extrapyramidal. The best known and most widely represented extrapyramidal disease is Parkinson’s disease. Quantitative EEG measures were frequently used to assess mainly the cognitive impact of the disease on the individual [35]. Some authors proposed the method as a sensitive predictor of the cognitive decline [36]. The method seems to discriminate, using different patterns of slowing with different topographies, between executive dysfunction and dementia, both characteristic for the disease [37]. The method was used to offer a tool for discriminating between Parkinson’s and Lewy body disease, with promising results [38]. Despite this extensive spectral EEG information, motor characteristics are not presented satisfactorily in the literature. The study of repetitive magnetic stimulation in Parkinson’s disease is wide and even got to the point of using the method as an add-on along with the pharmacological treatment, for symptomatic relief. Still, conduction studies are sparse, without clinical significance; the literature focuses mainly on the symptomatic use [39]. EMG studies in Parkinson’s disease are either showing no relevant difference with healthy controls [40], or are performed with other techniques, like surface EMG [41], without any information about turn-amplitude analysis.

Neuromuscular Diseases. Quantitative EEG assessment of the motor cortex is a field of continuous investigation, the main focus here being the functional network disruption [42]. Besides the primary motor dysfunction, there are other affected networks, such as attention switching [43], with consequences on motor planning. Both motor and non-motor networks show distinct interruptions on the spectral EEG, correlated with the main clinical features of the disease [44]. Transcranial magnetic stimulation is investigated for different purposes in motor neuron disease, both as a diagnostic tool to assess central motor conduction [45], and as an experimental method to alleviate enhanced excitatory activity [46]. Last, the EMG methods are widely used in the evaluation of motor neuron diseases, even as predictive methods for the evolution of the motor capabilities [47].

For the above presented pathologies, this combined method of evaluation, targeting the impact of clinical interventions on the stimulation of the plasticity of neurophysiological pathways is designed to determine the level of the correlation between different electrophysiological signals. In particular, these methods are assessing the activity of cortical to muscular structures involved in motor activities [48]. This work is part of an evaluation of the management of the neurological patient in order to improve the efficiency and reduce the societal cost of rehabilitation programs.

This paper proposes an inaugural exploratory study investigating the interchangeability of an experimental robotic rehabilitation device system and classical physical therapy. The Materials and Methods section presents a multimodal neurophysiological assessment of the motor system-quantitative EEG, motor conduction times and turn/amplitude analysis as detailed in Section 2. Section 3 lists the results of the study and Section 4 discusses and analyzes the obtained data. The conclusions of the study are drawn in Section 5.

## 2. Materials and Methods

### 2.1. ASPIRE and ParReEx Rehabilitation Robotic Systems

**Figure 1:**
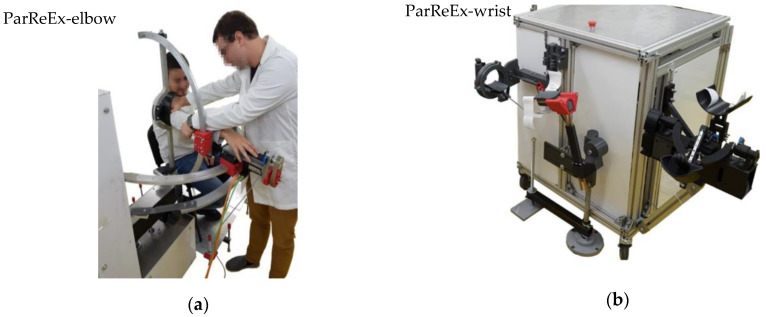
The modular robotic systems for upper limb rehabilitation: (**a**) ASPIRE; (**b**) ParReEx.

As mentioned, both ASPIRE [49] and ParReEx [50] robotic systems are controlled using a single central control interface. The two robotic systems were designed for the upper limb rehabilitation with their particularities detailed below:

- ASPIRE (Figure 1a) is a parallel robotic system with three Degrees of Freedom (DOF) based on a spherical architecture designed for the shoulder rehabilitation and targets the following motions: (a) shoulder flexion/extension and adduction/abduction; (b) forearm pronation/supination. The architecture of the mechanism allows a generalized movement on the sphere surface, which has the following advantages: (i) it enlarges the universality degree since the patient is positioned with his shoulder near the center of the sphere, and the anthropometric variations do not impose a problem; (b) the robotic system enables the definition of a wide range of exercises with various amplitudes and un-constrained working volume (including both simple and combined movements) which increase the shoulder mobility through interactive trajectories [18,19,51];
- ParReEx (Figure 1b) is a parallel robotic system which consists of two independent (decoupled) modules: (a) ParReEx-elbow with two DOF, designed for the elbow flexion and pronation/supination motion; (b) ParReEx-wrist with two DOF, designed for the wrist flexion/extension and adduction/abduction. Both ParReEx modules are able to perform simple and complex exercises based on interactive trajectories [20,21,52].

### 2.2. The Evaluation Protocol

The study protocol was approved by the Ethics Committee of the Municipal Clinical Hospital Cluj-Napoca (SCMCJ), being in accordance with the Helsinki principles for biomedical research; all patients signed an informed consent. The study started in October 2019 and lasted until December 2019. A total of 23 patients (12 men and 11 women) were enrolled, all inpatients of the Neurology Department of the SCMCJ. A common characteristic of the participants was upper limb motor deficit, but plegic patients were not included. Patients with excessive spasticity, which did not allow the upper limb joint movements, were excluded. Demographic data and age-dependent difference analysis is shown in Table 1.

Three patient groups were formed, according to their pathology. The first group, referred as Vascular, consisted of patients with ischemic stroke and included six men and six women with variable degree of motor deficit. Stroke patients were with chronic ischemic stroke, by definition. Right brachial motor deficit benefited from robotic rehabilitation; left brachial motor deficit performed the rehabilitation sessions with the physical therapists. A second group consisted of patients with extrapyramidal pathology, named Extrapyramidal, at the end all being diagnosed with Parkinson’s disease, and included six patients, three women and three men. Motor disturbances were bilateral; physical therapy for the right upper limb was made by the robot and for the left by the physical therapist, to have an objective comparison method. The two sides were their own controls. A third group was formed by patients with neuromuscular pathology, named Neuromuscular (motor neuron disease-amyotrophic lateral sclerosis–5). The patients included in this group were two females and three males. To summarize: in the case of the Vascular group. we had 6 patients with left, and 6 patients with right hemiparesis, a total of 12 records, 6 with robotic and 6 with physiotherapist rehabilitation, the deficit being unilateral. For the Neuromuscular and Extrapyramidal groups, there were 5 and 6 patients, respectively, but in these cases, both limbs were affected. For these groups we had 10 and 12 records, but 5 or 6, respectively, were right upper limbs, trained by the robot and 5 or 6 were left upper limbs, rehabilitated by the therapist. Therefore, for each individual group, there are 10 to 12 records, a total of 34 records, taken from 23 patients. The physical therapy program was provided for the right upper limb with the help of the modular robotic system, and for the left upper limb by the therapist. For Extrapyramidal and Neuromuscular patients, the debut of symptoms is chronic. We enrolled patients still able to move their upper limbs. Both the robots and the therapists performed the same protocol, for one exercise cycle. Only passive physical therapy was used, using the same protocol and exactly the same exercises, carried out on each patient of a subgroup, regardless if the rehabilitation was done by the robot or the therapist, as shown below in Table 2.

On enrolling, patients were informed about the study and subsequently signed the informed consent. The baseline multimodal neurophysiological evaluation was performed. To establish whether the rehabilitation method, through the proprioceptive signaling and afterwards parieto-frontal stimulation has any effect on the motor system, we used a high density, standardized EEG cap with 128 electrodes and a CareFusion NicOne EEG, with qEEG facility. Electrodes over the motor zone were selected. Electrode impedance levels were kept under 5 kΩ. The EEG amplifiers had a band-pass from 0.5 to 40 Hz. Twenty minutes of EEG trace were recorded. During the evaluation, the eyes of the patients were kept closed. A total of 60 s of artifact free epochs were selected, with an average length of 2–2.5 s. On the obtained epochs, Fast Fourier Transformation was performed, and frequency values were then converted into relative power, the percentage of total power within each frequency band, for each investigated channel. The highest power density peak was also evaluated, for both activity and frequency.

The link from the motor cortex to the effectors is established by the central motor tracts, roots, plexuses and the peripheral nerves. To evaluate whether repetitive exercises might have an effect on conduction, total, peripheral and central motor conduction time measurements were carried out. The stimulus was delivered over the motor cortex using an R20 MagVenture system with an MMC-40 coil (provided by the Nexus Medical Association) and for the recordings on the target muscle, a 4 channel EMS Surpass EMG/ENG/EP system. The intensity was around 65–70%, to overcome the resting motor threshold, and the target muscle was for almost every patient the abductor pollicis brevis, and for those with atrophy of this muscle, the abductor digiti minimi.

Patients were evaluated for the effectors of the motor system, the muscles, using turn/amplitude analysis and needle EMG. Easily reachable target muscles, like extensor carpi radialis, were examined, assessing the changes of turns/amplitude values, in order to characterize the state of motor units, both as a result of the influence of the peripheral and the central pathological process. Thereafter, patients were retested, in order to determine the impact of rehabilitation, both in terms of physical therapy and in relation to robotic recovery. A four-channel Surpass EMG device was used to examine the extensor carpi radialis muscle. The patient was asked to extend the wrist with graded muscle contraction until a maximum of 5 kgf, measured with a dynamometer. The maximal force of extension at the initial examination was noted. The turn/amplitude values were recorded at maximum contraction. The final examination (after 7 days, twice a day physical therapy/or robotic rehabilitation) was performed at the same force.

**Figure 2:**
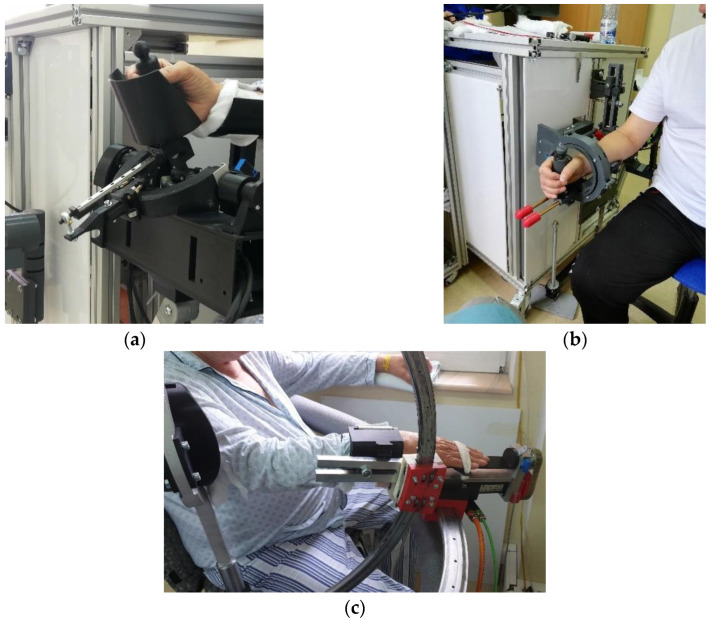
Images with patients during rehabilitation exercises with: (**a**) ParReEx wrist; (**b**) ParReEx elbow; (**c**) ASPIRE.

After the baseline assessments, a 7 day, 2 cycle/day rehabilitation program was implemented (see Table 1) to each patient, either by the modular robotic system (Figure 2) or therapist. A second examination, with all the mentioned tests, was made at the end of the 7 day physical therapy sessions.

All the obtained data was gathered in databases and pre-processed using MS Excel, and afterwards the statistical analysis was performed using IBM SPSS Statistics 20. After the descriptive statistics, the chosen method was non-parametric testing, given the low number of participants, and since each person was its own control, the Wilcoxon Matched Pairs Signed Ranks Test was used. The exception from this is the case of the Vascular group, where there are different individuals with left hemiparesis as a control group. In this case the Mann–Whitney U test for independent variables was used. Regardless the test, the significance threshold was *p* < 0.05.

## 3. Results

As previously stated, the neurophysiological tests were carried out before and after the physical therapy. Several data rows were gathered for each parameter, and before any further test was carried out, each set of values was tested for normality, using the Kolmogorov–Smirnov test (not presented here), in order to approximate the best evaluation method. All groups showed normal distribution.

The first test was the electroencephalographic recording. Since half of the patients were treated using the modular robotic system, and half by physical therapists, the first step of the evaluation was to use the Mann–Whitney U test for two independent samples to see if there was any difference between the two approaches. Virtually all evaluations were non-significant (see Table 3 below), except for the peak current density after one week of physical therapy, which was higher (marginal significance) in the case of patients treated by the therapist versus the robotic rehabilitation.

**Figure 3:**
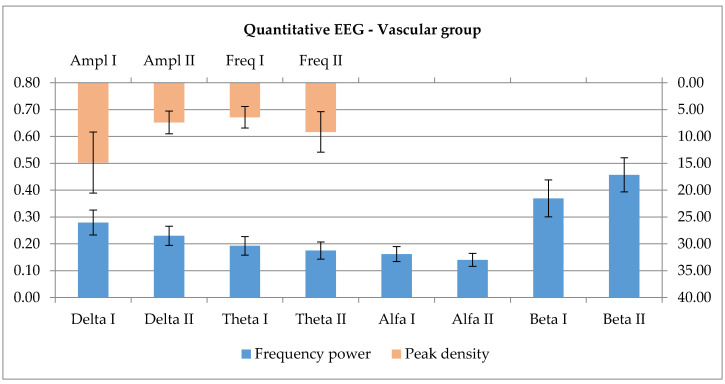
The evolution of frequency power representation over the motor zone and the changes in amplitude and frequency for the highest represented peak after 7 days of training.

Furthermore, since there was only one marginally significant difference between the two sides, the current density–relative power of each frequency band was investigated, but with the use of extended groups and 12 data entries for each parameter (both robotic and physical therapist rehabilitation). In the case of the Vascular group, there was a slight graphical tendency, but no statistical significance (p_delta_ = 0.424, p_theta_ = 0.905 and p_alpha_ = 0.196) towards the reduction of the lower frequencies (delta, theta and alpha) and an increase of the high frequencies’ representation (p_beta_ = 0.367). The highest peak of the represented frequencies was further investigated, showing a slight reduction of the amplitude and frequency elevation (Figure 3). Again, the relationship is not significant (p_Amp_ = 0.272 and p_Freq_ = 0.504). Figure 3 presents the parameters at the beginning and the end of the 7 day therapy session.

The next electrophysiological test targeted the conduction to the effectors and consists of the total (TMCT), peripheral (PMCT) and central motor conduction time (CMCT). There was no relevant difference between the two rehabilitation methods, right and left side; Table 4 presents the *p* values (Mann–Whitney U test).

**Figure 4:**
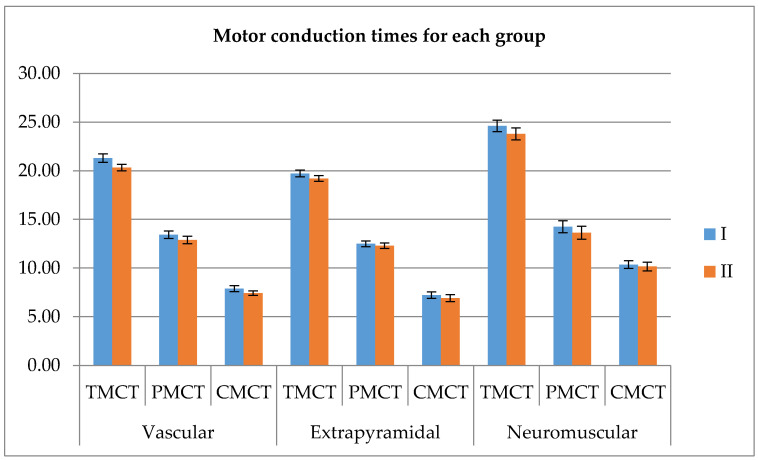
The total, peripheral and central motor conduction time before and after the 7 day rehabilitation process.

On the other hand, the overall differences are significant for the evaluation taken at the first and second visits. The total, peripheral and central conduction time show significant changes (p_TMCT_ = 0.002, p_PMCT_ = 0.005, p_CMCT_ = 0.028, Mann–Whitney U test for independent samples). Figure 4 presents the mean values in *ms* of the total, peripheral and central motor conduction times, and the error bars represent the standard error of means.

The last electrophysiological evaluation targeted the effector, the peripheral muscle. The extensor carpi radialis muscle of each participant was assessed, using turns/amplitude analysis, the main recorded parameters being the interval, amplitude, turns, ratio, activity and root mean square (RMS). The two sides are without real difference, between the rehabilitation therapy carried out by the therapist and by the robot, at least from this point of view (Table 5).

**Figure 5:**
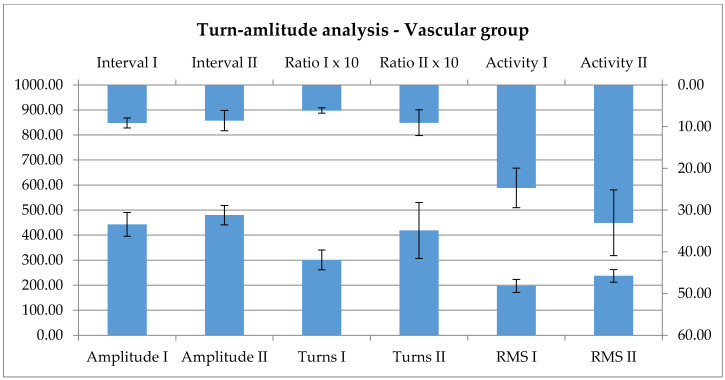
The parameters of the interference pattern analysis show a non-significant, only visual tendency towards increase as an effect of the therapy.

According to this, combined groups were formed, and evaluated the number of turns and their amplitude, the root mean squared and activity values, at the same force of the contraction, which was quantified using dynamometry. There is a clear increase of each value after the 7 day long increased activity, showing a higher recruitment, but also a higher activity level of each motor unit action potential of the evaluated area. Although the effects are not significant, there is a visual tendency towards increase, as it is shown in the below graphic (Figure 5).

The next evaluated group was the Extrapyramidal group. All participants were diagnosed with Parkinson’s disease. Similarly with the vascular group, the first neurophysiological assessment was the EEG. When evaluating separately, there was no significant difference between the left and right side (Table 6), excepting, here also, for the peak current density after the one week rehabilitation, a marginal value.

**Figure 6:**
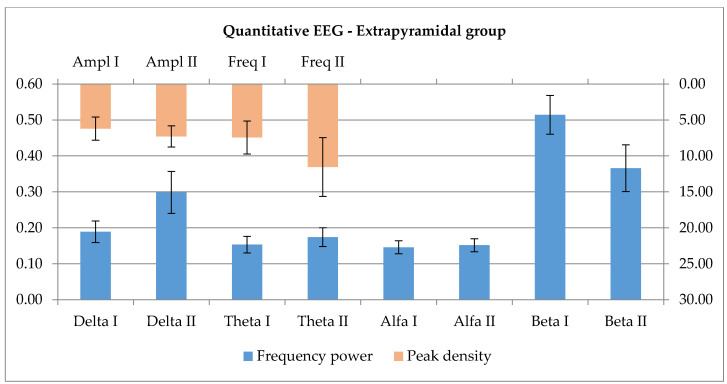
Relative power findings show an increase of slow activity in parallel with a decrease in fast activity in the Extrapyramidal group. The highest peak presented an increase in both amplitude and frequencies after the 7 day rehabilitation program.

Since there is virtually no significant change between the two sides–the existing marginal significance probably will lose its strength with the increase of the number of cases, the same method of forming extended groups of 12 entries each was applied, using all available data for every single patient. The trace is modulated by the 7 day long intensive proprioceptive signaling towards an increase of slower activities (p_delta_ = 0.126, p_theta_ = 0.610, p_alpha_ = 0.504), and a decrease of higher frequencies (p_beta_ = 0.099), these being increased basally. Although visually there’s a clear tendency, there is no statistical significance (Wilcoxon signed rank test). When investigating the peak, the amplitude showed a moderate tendency for increase, and also the registered frequency increased–see Figure 6, both changes non-significant (p_Amp_ = 0.583, p_Freq_ = 0.556, Wilcoxon).

The conduction times were performed as presented for the vascular group, the first evaluation being the between sides comparison, without significant differences (Table 7).

Since differences were without significance in the sidewise comparison, extended groupswere formed, using all available measurements. Further, the parameters gathered at inclusion versus the data obtained after the rehabilitation were compared, the results showing little, but significant change for TMCT and PMCT, not CMCT in the extended groups (p_TMCT_ = 0.011, p_PMCT_ = 0.017, p_CMCT_ = 0.165, Wilcoxon). Below, there are the graphical representations showing the relations (Figure 4).

The peripheral effector was tested using IPA. First the between sides differences were evaluated, Table 8 shows a general lack of significant differences, excepting the activity for the second evaluation, with marginal significance.

**Figure 7:**
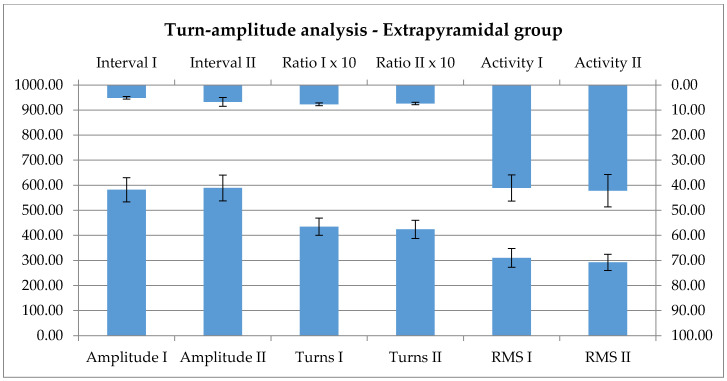
Interference pattern analysis parameters are notably not influenced by the rehabilitation.

The method presented for the vascular group was used for further testing. The same lack of effect is seen on the turns-amplitude analysis; here, the peripheral effector is not modified significantly, as an effect of the rehabilitation. Below, in Figure 7, there is the comparable graphical representation of the data at the first and second visit.

The last group was represented by Neuromuscular diseases. There were no differences between the two sides for the evaluated qEEG values, as shown in Table 9.

**Figure 8:**
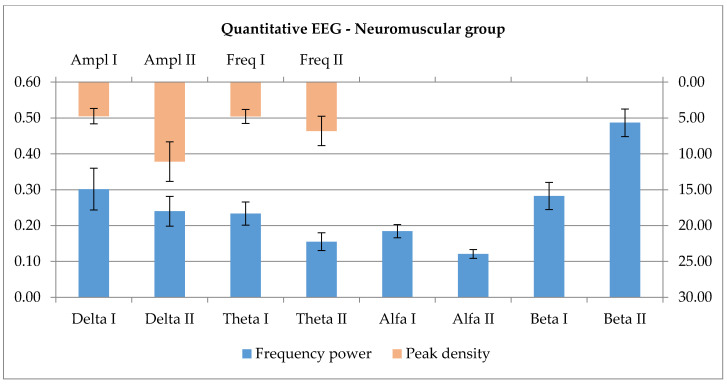
The representation of slower qEEG frequencies are diminished, and high frequency activity seems to increase after the 7 day continuous exercises. The highest peak shows an increase in amplitude and frequency, a more robust representation of higher frequency range after exercise.

All values were used for the further statistical analysis, combined groups being formed. The qEEG evaluation shows a pattern of activation with significant changes for the higher frequency domains (p_Beta_ = 0.003), with concomitant reduction of the lower frequency domains, in a significant degree for alpha (p_Alpha_ = 0.02) and marginal for theta (p_Theta_ = 0.091), and only a visual tendency for delta (p_Delta_ = 0.209). For the best represented domain, there is a significant increase of the amplitude after the one-week rehabilitation (p_Ampl_ = 0.023). The frequency shows no relevant change after the physical therapy (Figure 8).

The conduction in the motor system in case of the Neuromuscular group shows no significant sidewise difference (see Table 10).

Given the above result, all values were evaluated and significant post-rehabilitation reductions were obtained for TMCT and PMCT (p_TMCT_ = 0.005, p_PMCT_ = 0.008), see Figure 4.

Considering the peripheral effectors, there is a significant difference between the left and right side, in the case of post-rehabilitation evaluation for some of the parameters, the interval, amplitude, the activity and the RMS (see Table 11).

All these parameters show a reduction as an effect of the rehabilitation, but the reduction seems to be more pronounced, and significant in case of robotic rehabilitation.

**Figure 9:**
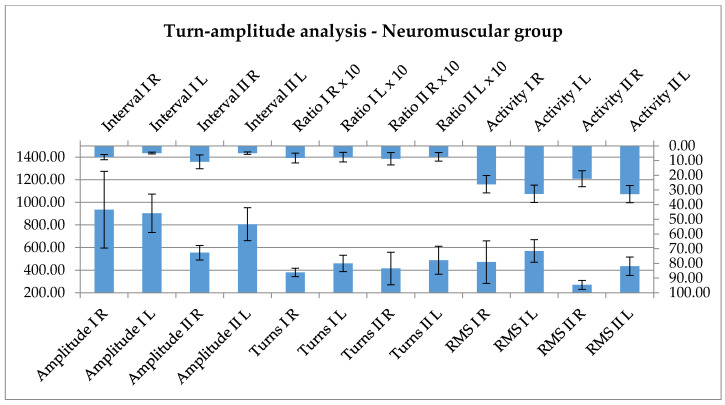
The parameters of the interference pattern analysis show a tendency for decrease after the rehabilitation.

Since there were differences during the sidewise evaluation, the further analysis was performed using each data row independently. Figure 9 shows the tendencies towards, there were no significant changes, either with robotic or human approach.

## 4. Discussion

The above presented results offer a preliminary idea about what functional changes might produce the rehabilitation for the targeted pathologies. In the below presented sections, data are grouped according to pathology. Significant differences were rarely seen, given the small number of participants; tendencies are discussed instead, to point out possible outcomes for the moment when the study will reach a wider population and not only be on an exploratory level.

Ischemic stroke produces a typical pattern of damage in the CNS, characterized by central regions–vascular territory, characterized by cell death, and peripheral penumbra zones, able to recover to various points, if circumstances favorable. In case of the Vascular group, the first observation was the difference between the peak current density, which shows a marginally significant value for the classical physical therapy versus the robotized approach. Since there was a really thin difference, it was considered as non-significant; still, what if a higher number of cases validates this relation? Under that circumstance, it raises a question as to whether human contact can further enhance the activation of the damaged brain area, or if there is no difference compared with the robot. The slight reduction of the amplitude and frequency elevation probably means wider activated brain areas as a consequence of the therapy. This requires further research, and there are no reliable data in the literature in this regard. The EEG recordings and spectral power changes show tendencies towards reduction of the lower frequencies (delta, theta and alpha) and an increase of the high frequencies representation (beta). In the literature, the main feature of central lesions is increased slow activity representation [23,24]. From this point of view, if the rehabilitation produces as a central functional expression an increase of the higher frequencies, this underlines a beneficial effect, regardless of whether the rehabilitation is conducted by a physical therapist or by a robotic device.

The literature considers conductibility measures as a valuable tool [28] for the evaluation of post-stroke patients, and even a good predictor for recovery [29,30]. Consistent with the mentioned data, the present research shows a significant reduction of the total, peripheral and central conduction time when comparing the initial and post-rehabilitation data, regardless if the protocols were applied by the therapist or by the robotic device. More extended study groups might even allow us to validate the method.

The EMG data, and among these, the turn-amplitude analysis evaluations, are sparse in the literature [31]; still, one can find sporadic information about changes during a rehabilitation process [32], consistent with the recovery of activation of a wider number of motor units in the periphery [34]. Data presented above seems to be in line with these: there is an increase of the number of turns and their amplitude, the root mean squared and activity values after the 7 day long increased activity. The relationship is only a tendency, not significant; still, this might become more expressed by increasing the number of participants. This means that there is a higher recruitment and a higher activity level. By activating wider central areas, the peripheral connectivity is also showing a positive progression, this being expressed by the recovery of function in a wider number of motor units. Since the central effect was not influenced by the applied rehabilitation method, it was also expected not to have such an effect in the periphery. Indeed, there is no difference between the activation patterns for the two methods.

For the Extrapyramidal group the premises are different. The activation/modulation of motor activity is realized through the fine-tuned co-work of the direct and indirect pathways linking the motor cortex to the basal ganglia system. Excitatory influences initiated by the cortex act on the striatum, which gets to inhibit the internal globus pallidus (GPi) through a GABA-ergic input modulated by dopamine release from the substantia nigra (D1 receptors). As a consequence, the inhibition of the GPi on the ventro-lateral and ventro-anterior nuclei of the thalamus is reduced and there will be an increased excitatory feed-back loop from the mentioned nuclei to the cortex. This is the direct pathway which, in the case of reduction of dopamine availability, results in reduction of tonic excitatory influences on the cortex, reduction of activities towards initiation of movement. The indirect pathway starts with the cortico-striatal excitatory influences, but the dopamine release, through D2 receptor signaling, inhibits the external globus pallidus (GPe), which inhibits the subthalamic nucleus, so it will reduce the inhibition; this, in turn, has an overall excitatory effect on the GPi. The latter, as a consequence, acts by a more enhanced inhibition on the thalamic nuclei, which in turn reduce their excitatory influences on the cortex. Dopamine reduction promotes the indirect pathway. The current understanding of beta activity in the motor area is linked with grasping, reaching, attention processes and muscle contraction [53]. As presented in the introduction, different ranges of EEG slowing in the motor area, with various topographies [35] are mentioned as features of the disease [38]. During the experiments, a non-significant relation was found, but there is a tendency towards increase of the slower frequency domains and a reduction of the beta spectrum. When investigating the peak frequency, the amplitude showed a moderate tendency for increase, and also the registered frequency increased. In the prism of the above-mentioned data, the proprioceptive input seems to increase the excitatory cortico-striatal influence which, in a condition with reduced dopamine availability, further enhances the activity of the indirect pathway, and through this, the thalamic excitatory loop is inhibited. As a consequence, the question is raised if the used rehabilitation protocol has an overall beneficial effect on the patients or not. Further, a larger study is needed to properly analyze this relationship. The two methods show a marginally significant value for the peak current density, more decreased in case of rehabilitation with the physical therapist. The present research revealed no current explanation for this; one might expect an opposite effect, considering the cognitive impact of interacting with a human; the difference will probably be attenuated with an increase of studied cases.

Conduction studies are sparse in the literature. The above results show a significant reduction of TMCT and PMCT, but not CMCT, in the extrapyramidal groups, suggesting that this improvement of conduction is present independently, when using the method, regardless the disease. The effect is present for both the robotic rehabilitation and the therapy conducted by the physical therapist. By extending the tests on more subjects, might probably validate this effect.

The turn-amplitude analysis shows no significant changes after the 1 week rehabilitation, not even tendencies; otherwise, this is in accordance with the poor data of the literature [41].

Last, the Neuromuscular group’s data is discussed, in fact amyotrophic lateral sclerosis patients. The disease affects both central and peripheral motor neurons, and the in-between pathways, leading to the peripheral loss of muscle fibers as a consequence of progressive destabilization and destruction of motor units. The functional evaluation of the central nervous system reveals patterns of disruption of mainly motor networks [42]. By reduction of the cell population of the motor area, the potential generator layer of movement is also lost. A baseline preponderance of slow activities is to be expected. Rehabilitation, on other hand, might have a stimulating effect, with an increase of fast activities. The role of excessive stimulation is debatable, given the glutamate excitotoxicity, which is an important pathogenetic factor in the disease course [54]. The presented results reflect a pattern of activation with significant changes for the higher frequency domains, with concomitant reduction of the lower frequency domains, in a significant degree for alpha and marginal for theta, and only a visual tendency for delta. By knowing the underlying mechanisms, the effect probably is not beneficial. Still, the impact is not really disease-modifying, even if there is a significant increase of the best represented frequency domain.

The conduction studies show the same tendencies as for the previously mentioned two diseases: a significant reduction of both TMCT and PMCT with a tendency only for CMCT. This effect has to be based on the same stimulating effect seen before, and the lack of impact on CMCT might be caused by the direct affection of the cortico-spinal pathways. Evaluation of the latter became lately a widely used diagnostic tool [45].

EMG evaluation, turn/amplitude analysis also, is frequently used in the diagnosis of motor neuron diseases [47], with some prognostic valences also. It targets the peripheral effectors, which show significant changes during the disease course, fascicullations, atrophy and dysfunction. There are many unstable motor units: some lost forever, some still active, some under reinnervation. Physical exercise in theory potentially maintains the function, but probably acts only on still functionally intact motor units. The other undergoing processes—ongoing loss of fibers, reinnervation—might suffer an exhaustive effect. The results show this kind of tendency: a lack of peripheral activation. Instead, all the parameters of activity are decreasing; the reduction seems to be more pronounced and significant in case of robotic rehabilitation, where the stereotyped rehabilitation program is not taking into account the exhausting effect and is not adapted to the convenience of the patient yet. This raises the question if physical rehabilitation programs are beneficial for muscle function, or are only useful to maintain joint flexibility in motor neuron diseases.

The overall effect of the rehabilitation is towards a slight change, even in the case of robotic approach, regardless of the disease. In this situation, given the reduced availability of physical therapists, the role of robotic rehabilitation might become important and at least deserves the premises to extend the research to a higher number of patients, as the literature suggests [55].

The study has an important limitation, pointed already out in the discussion: the low number of cases. This issue might be solved by further investigations, extending the number of participants probably will lead to more relevant conclusions. This was a short, inaugural exploratory study, but with multimodal approach, which led to the fine-tuning of the rehabilitation devices and the formulation of new targets for a better clinical approach.

## 5. Conclusions

Overall, this multimodal neurophysiological evaluation allows to formulate three major conclusions: (i) there is little, if significant difference between robot-assisted or physical therapist performed rehabilitation therapy; (ii) the chosen neurophysiological tests are clearly influenced by rehabilitation, future studies might validate these as objective measures; (iii) the effect of the therapy on different pathologies is not necessarily positive: it is either beneficial, in case of vascular group or debatable/limited, in case of extrapyramidal and neuromuscular groups.

For the Vascular groups, a significant reduction of the total (peripheral and central) conduction time (after rehabilitation) was found, regardless if the protocols were applied by the therapist or by the robotic device. This result predicts patient recovery and validates (to a limit) the benefits of the robotic assisted rehabilitation paradigm. Furthermore, a significant reduction of TMCT and PMCT (but not CMCT) was also found in the Extrapyramidal and Neuromuscular groups (for both robotic rehabilitation and the therapy conducted by the physical therapist), an effect that needs further investigations to be validated. Further clinical studies are required to overcome the main limitation of this study (the low number of patients in the groups) in order to validate the robotic assisted rehabilitation outcomes using the novel robotic system in parallel with several improvements for the robotic structure in terms of ergonomics, user interface and interactive patient applications.

## Figures and Tables

**Table 1:** Demographic data showing the age and gender distribution, with no significant difference between the groups.

	Vascular	Extrapyramidal	Neuromuscular
Age			
Mean ± s.e.	75.92 ± 1.77	71.17 ± 4.13	66.00 ± 3.85
*p* (Kruskal-Wallis)	0.138		
Gender			
Male	50%	50%	60%
Female	50%	50%	40%

**Table 2:** The applied physical rehabilitation protocol.

Vascular Group	Extrapyramidal Group	Neuromuscular Group
Passive exercises of the upper limb, 2 times a day 10–12 repeats:	Passive exercises of the upper limb, 2 times a day 10–12 repeats:	Passive exercises of the upper limb, 2 times a day 8–10 repeats:
-phalanx flexion	-Pollicis flexion	-phalanx flexion
-finger flexion and extension	-phalanx flexion	-finger flexion and extension
-Radio-carpal joint flexion and extension	-finger flexion and extension	-Radio-carpal joint flexion and extension
-Radio-carpal joint rotation	-Radio-carpal joint flexion and extension	-Radio-carpal joint rotation
-forearm supination and pronation	-Radio-carpal joint rotation	-forearm supination and pronation with slight resistance
-elbow flexion	-forearm supination and pronation	-elbow flexion with 10–20% resistance
-shoulder flexion and extension	-elbow flexion	-stretching program, positioning in extension
-shoulder adduction and adduction	-shoulder flexion and extension	-shoulder adduction and adduction
-shoulder rotation	-shoulder adduction and adduction	-shoulder rotation against reduced resistance
	-shoulder rotation	

**Table 3:** The *p* values obtained after sidewise testing (Mann–Whitney U Test) for the qEEG parameters. Marginal significance for current density in the Vascular group.

Left vs. Right (Mann-Whitney U Test)		Delta		Theta		Alpha		Beta		Peak			
										Density (mV2)		Freq. (Hz)	
		I	II	I	II	I	II	I	II	I	II	I	II
*p*	Vascular	0.22	0.75	0.21	0.4	0.4	0.25	0.75	0.6	0.75	0.05	0.14	0.75

**Table 4:** The between side evaluation for motor evoked potentials (MEP) parameters; no significant *p* values.

Left vs. Right	TMCT_I	TMCT_II	PMCT_I	PMCT_II	CMCT_I	CMCT_II
*p*	0.75	0.47	0.94	0.94	0.47	0.94

**Table 5:** The between side evaluation for interference pattern analysis IPA parameters; no significant *p* values.

Left vs. Right (Mann-Whitney U Test)	Interval (ms)		Amplit. (µV)		Turns (1/s)		Ratio		Activity (%)		RMS (µV)	
	I	II	I	II	I	II	I	II	I	II	I	II
*p*	0.63	0.75	0.87	0.52	0.75	0.75	0.81	0.34	0.75	0.75	0.75	0.87

**Table 6:** The *p* values obtained after sidewise testing (Wilcoxon) for the qEEG parameters. Marginal significance for current density in the Extrapyramidal group.

Left vs. Right (Wilcoxon)		Delta		Theta		Alpha		Beta		Peak			
										Density (mV2)		Freq. (Hz)	
		I	II	I	II	I	II	I	II	I	II	I	II
*p*	Extrapyramidal	0.60	0.20	0.59	0.40	0.50	0.68	0.35	0.17	0.25	0.05	0.08	0.27

**Table 7:** The *p* values obtained after sidewise testing (Wilcoxon) for the MEP parameters.

Left vs. Right (Wilcoxon)	TMCT I	TMCT II	PMCT I	PMCT II	CMCT I	CMCT II
*p*	0.67	0.50	0.34	0.42	0.69	0.22

**Table 8:** The *p* values obtained after sidewise testing (Wilcoxon) for the IPA parameters. Marginal significance for Activity.

Left vs. Right (Wilcoxon)	Interval (ms)		Amplitude (µV)		Turns (1/s)		Ratio		Activity (%)		RMS (µV)	
	I	II	I	II	I	II	I	II	I	II	I	II
*p*	0.35	0.92	0.17	0.17	0.60	0.75	0.17	0.34	0.92	0.05	0.34	0.46

**Table 9:** The *p* values obtained after sidewise testing (Wilcoxon) for the qEEG parameters.

Left vs. Right (Wilcoxon)		Delta		Theta		Alpha		Beta		Peak			
										Density (mV2)		Freq. (Hz)	
		I	II	I	II	I	II	I	II	I	II	I	II
*p*	Neuromuscular	0.21	0.59	0.60	0.26	0.14	0.68	0.35	0.47	0.14	0.35	1.00	0.20

**Table 10:** The *p* values obtained after sidewise testing (Wilcoxon) for the MEP parameters.

Left vs. Right	TMCT I	TMCT II	PMCT I	PMCT II	CMCT I	CMCT II
Neuromuscular	0.25	0.89	0.28	0.07	0.46	0.24

**Table 11:** The *p* values obtained after sidewise testing (Wilcoxon) for the IPA parameters in case of the Neuromuscular group.

Left vs. Right (Wilcoxon)	Interval (ms)		Amplit. (µV)		Turns (1/s)		Ratio		Activity (%)		RMS (µV)	
	I	II	I	II	I	II	I	II	I	II	I	II
*p*	0.35	0.046	0.75	0.03	0.35	0.07	0.92	0.60	0.60	0.046	0.60	0.03
